# Cochlear implant electrode impedance subcomponents as biomarker for residual hearing

**DOI:** 10.3389/fneur.2023.1183116

**Published:** 2023-05-23

**Authors:** Stephan Schraivogel, Philipp Aebischer, Stefan Weder, Marco Caversaccio, Wilhelm Wimmer

**Affiliations:** ^1^Hearing Research Laboratory, ARTORG Center for Biomedical Engineering Research, University of Bern, Bern, Switzerland; ^2^Department of ENT—Head and Neck Surgery, Inselspital, Bern University Hospital, University of Bern, Bern, Switzerland; ^3^Department of Otorhinolaryngology, TUM School of Medicine, Klinikum Rechts der Isar, Technical University of Munich, Munich, Germany

**Keywords:** hearing preservation monitoring, cochlear trauma, electrode-tissue interface, follow-up, objective measure

## Abstract

**Introduction and objectives:**

Maintaining the structural integrity of the cochlea and preserving residual hearing is crucial for patients, especially for those for whom electric acoustic stimulation is intended. Impedances could reflect trauma due to electrode array insertion and therefore could serve as a biomarker for residual hearing. The aim of this study is to evaluate the association between residual hearing and estimated impedance subcomponents in a known collective from an exploratory study.

**Methods:**

A total of 42 patients with lateral wall electrode arrays from the same manufacturer were included in the study. For each patient, we used data from audiological measurements to compute residual hearing, impedance telemetry recordings to estimate near and far-field impedances using an approximation model, and computed tomography scans to extract anatomical information about the cochlea. We assessed the association between residual hearing and impedance subcomponent data using linear mixed-effects models.

**Results:**

The progression of impedance subcomponents showed that far-field impedance was stable over time compared to near-field impedance. Low-frequency residual hearing demonstrated the progressive nature of hearing loss, with 48% of patients showing full or partial hearing preservation after 6 months of follow-up. Analysis revealed a statistically significant negative effect of near-field impedance on residual hearing (−3.81 dB HL per kΩ; *p* < 0.001). No significant effect of far-field impedance was found.

**Conclusion:**

Our findings suggest that near-field impedance offers higher specificity for residual hearing monitoring, while far-field impedance was not significantly associated with residual hearing. These results highlight the potential of impedance subcomponents as objective biomarkers for outcome monitoring in cochlear implantation.

## 1. Introduction

With more than 1 million implanted devices worldwide, the cochlear implant (CI) is the most successful treatment for patients suffering from partial to complete deafness (1). CI candidacy has been relaxed to include patients with residual acoustic hearing. For these patients in particular, preservation of residual hearing and structural integrity of the cochlea during and after CI surgery are important goals to improve hearing outcomes (2–4). However, a considerable number of patients lose their residual hearing either during or after cochlear implantation [mean hearing preservation after 1 month: 82%, 6 months: 76%, and 12 months or more: 69%; (5)]. Intraoperative loss of residual hearing is associated with intracochlear trauma (6, 7) caused by the insertion of the electrode array. Postoperative hearing loss is related to fibrous tissue and new bone formation in the cochlea (8, 9). Another reason for postoperative hearing loss could be inflammatory/foreign body response to the platinum-iridium electrodes and the surrounding silicon carrier (10, 11).

Reliable biomarkers are needed for continuous monitoring and adaptation to changes in residual hearing, especially for electric acoustic CI recipients (12). Impedance telemetry allows measuring electrical impedances and has been performed since the first CIs (13). Impedances are in focus for several purposes, including insertion outcome monitoring [e.g., insights into the cochlear microenvironment around CI electrode contacts (14), fibrous tissue growth (15, 16), or inner ear pathologies (17)], electrode position monitoring within the cochlea (18–21), or continuous monitoring of electrode contact integrity with remote apps (22).

In an exploratory study, we demonstrated the association between clinical impedances and residual hearing (23). The term clinical impedance encompasses a mixed quantity that can be divided into contributions from the electrode-electrolyte interface and nearby bulk resistance (near-field impedance) and electrical resistance through biological tissue from the stimulating electrode to the ground electrode on the implant body (far-field impedance) (21, 24). Based on these findings, we hypothesize that impedance subcomponents could yield more specific data to monitor hearing performance in the follow-up after cochlear implantation. Therefore, the aim of this study is to evaluate the association between residual hearing and impedance subcomponents of a previously published approximation model (21) in a known collective from a previous study (23).

## 2. Methods

### 2.1. Study design

We performed a refined retrospective analysis of residual hearing and impedance subcomponent data from the same cases as previously reported in Wimmer et al. (23). The study was approved by our local institutional review board (ID 2019-01578). The study included patients who met the following criteria: (a) underwent cochlear implantation at our center between January 2009 and June 2021, (b) having an electrode array from MED-EL (Innsbruck, Austria), (c) having a low-frequency residual hearing (i.e., between 0.125 and 1 kHz) of at least 5 dB HL, and (d) having a postoperative follow-up of at least two pure tone audiograms and corresponding impedance telemetry recordings within a minimum of 6 months.

### 2.2. Residual hearing data

According to the standard clinical procedure, we used a clinical audiometer along with insert earphones or a headphone to measure pure tone air conduction hearing thresholds in dB hearing level (HL) at seven frequencies (0.125, 0.25, 0.5, 1, 2, 4, and 8 kHz). All audiological measurements were performed in an acoustic chamber. We calculated residual hearing as the absolute difference between the maximum detectable hearing levels by the audiometer (i.e., 90 dB HL at 0.125 kHz, 110 dB HL at 0.25 kHz, and 120 dB HL for the remaining frequencies) and the measured hearing thresholds at the corresponding frequency. Low-frequency Pure tone average (PTA) was computed as the mean of residual hearing at frequencies between 0.125 and 1 kHz.

### 2.3. Impedance telemetry data

Impedance telemetry data were recorded using the standard clinical protocol of the manufacturer's telemetry software (MAESTRO, MED-EL, Austria) and were recorded in the same sessions as pure tone audiometry. In addition, to analyze the by-case progression of impedances over time, we retrieved all available impedance telemetry data from the day of implantation up to a maximum follow-up time of 100 months. The clinical electrode impedance can be subdivided into two components: near-field and far-field impedance. The near-field impedance is associated with the local cochlear microenvironment around the stimulating electrode. The far-field impedance provides information about the electrical return path through biological tissue from the stimulating electrode to the ground electrode on the implant body (24). We estimated the far-field impedance, also called tissue resistance, using bivariate spline extrapolation of the impedance telemetry recordings as established in previous work (20, 21). Subsequently, near-field impedance was defined as the difference between clinical impedance and far-field impedance. We excluded data from extracochlear electrodes that were identified from computed tomography (CT) scans (18 electrodes from six cases, see demographics, Supplementary Table 1). In addition, we excluded samples of a single electrode from further analysis if an open circuit was detected by the manufacturer's telemetry software (at 30 electrodes from eight cases).

### 2.4. Computed tomography data

We used the open-source software 3D slicer (25) to measure the cochlear base length and width (26, 27) in the preoperative CT scans and calculated the cochlear duct length without the hook region length [i.e., starting at an angular insertion depth of 0° (28–30)]:


(1) $$\begin{array}{l} CDL\left(0^{{}^{\circ}}\right)=1.71\times \left(1.18\;A_{OC}+2.69\;B_{OC}-\sqrt{0.72\;A_{OC}\;B_{OC}}\right)+0.18, \end{array}$$

where *A*_*OC*_ and *B*_*OC*_ are the cochlear base length and width subtracted by 1 mm, respectively.

### 2.5. Statistical analysis

We used a linear mixed-effects model to assess the relationship between near and far-field impedances and residual hearing. For each case, repeated measurements were taken on different days after cochlear implantation. To account for the dependence of observations, we used a case-level random intercepts model, which allows the intercept to vary per case, i.e., for each case a unique effect is added to the overall intercept. Random slopes of impedance subcomponents allowed different by-case slopes for the independent variables.

First, we created a model with residual hearing (in dB HL) as the dependent variable and impedance subcomponents (in kΩ) as the independent variables. In addition, the follow-up time (in months), the implanted side (left vs. right), the gender (female vs. male), the electrode array type (FLEX24, FLEX28, FLEXSoft, or Standard), the cochlear duct length (in mm), and age (in years) were included as fixed effects. We added interaction terms between the impedance subcomponents and follow-up time, as impedance changes are associated with follow-up time (23). We assumed correlations between random slopes for near-field and far-field impedances [i.e., random effects were specified as *(near-field impedance* + *far-field impedance* ∣ *case-level)*]. We compared the model depending on impedance subcomponents to a second model depending on the total clinical impedance and the same fixed effects as in the first model.

The models were compared in terms of the Akaike information criterion (AIC) and the Bayesian information criterion (BIC), the amount of explained variance of the dependent variable by the independent variables (using the coefficient of determination *R*^2^), and the amount of explained variance due to grouping [using the Intraclass correlation coefficient (ICC)]. The statistical analysis was performed in the RStudio environment (31) using the lme4 package (32).

## 3. Results

### 3.1. Demographics and word recognition

We considered a total of 704 recordings in our center's database between January 2009 and June 2021. Of these, 42 patients met the inclusion criteria. The patients' age ranged from 10 to 80 years (median age 57 years). Of the 24 female and 18 male patients, there were 21 left and right-sided implantations, respectively. With 26 cases, the majority were implanted with a FLEX28 electrode array. Partially inserted electrode arrays were present in six cases (1 case each with 1 and 2 extracochlear electrodes, three cases with 3, and 1 case with 6). After activation for 6 months, the median word recognition score was 100% (interquartile range [90, 100]%) for the German Freiburg numbers test and 58% (interquartile range [40, 73]%) for the German Freiburg monosyllabic word test (see Supplementary Table 1).

### 3.2. Impedance subcomponents progression over time

**Figure 1:**
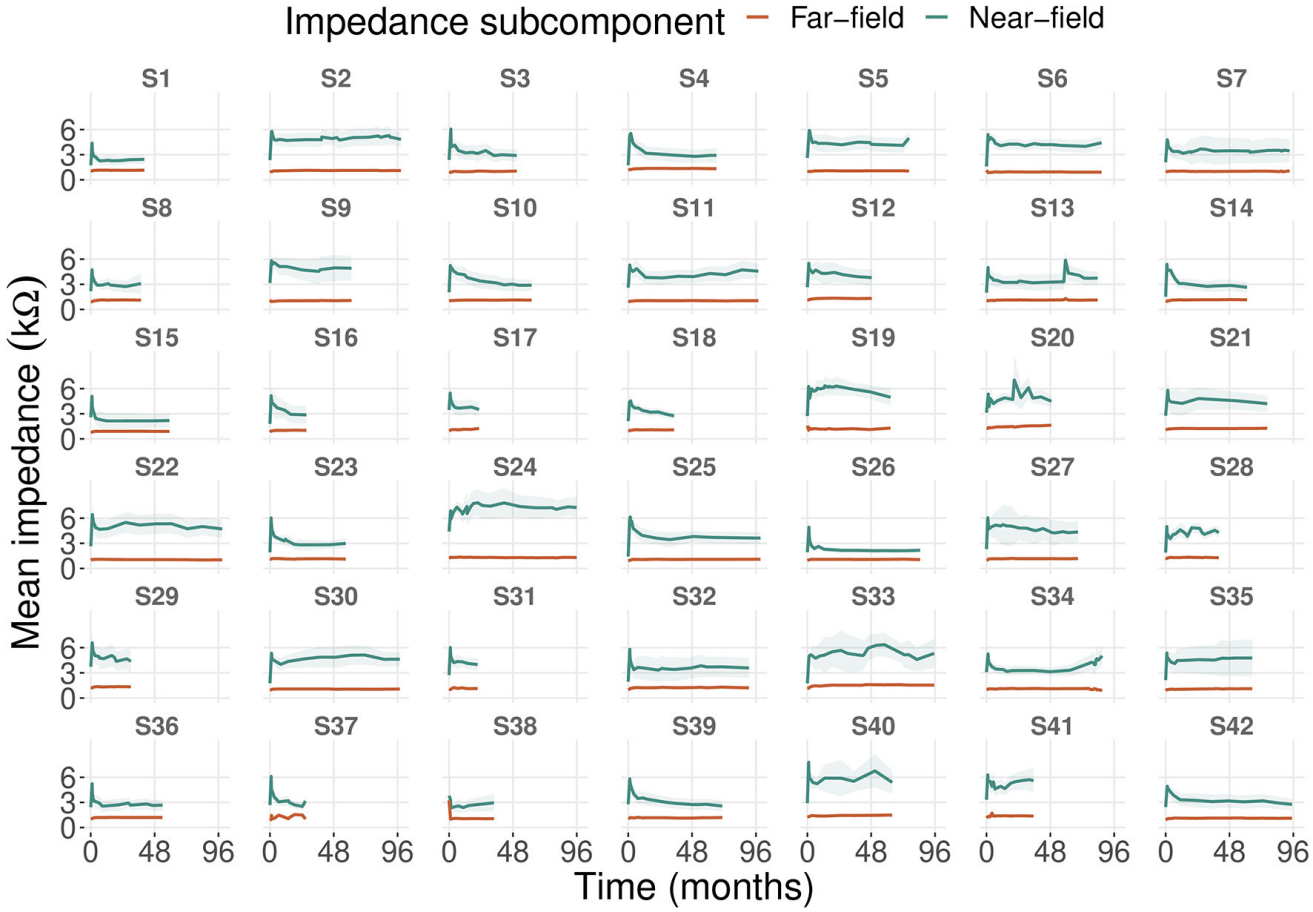
Progression of mean near-field and far-field impedances of 42 cases including all electrodes. The shaded area represents the 95% confidence interval of the mean.

We analyzed impedance subcomponents progression based on 635 recordings with a mean follow-up time of 24 months (ranging from 21 to 99 months). Mean far-field impedances were more stable over time compared to mean near-field impedances (case-level standard deviations of ~0.1–0.7 kΩ and 1–2.4 kΩ, respectively; Figure 1). After the date of implantation, near-field impedances increased strongly until the first activation session (first month) and stabilized after ~6–12 months. These dynamics were most pronounced at the most apical electrode and decreased toward the round window until electrode 10 (see Supplementary Figure 1).

Far-field impedances did not show these dynamics. For the two most basal electrodes, near-field impedances increased over time starting 3 months after implantation. In contrast, far-field impedances at the two most basal electrodes already began to increase between the date of implantation and activation (first month). In general, mean levels of far-field impedances were higher at the apical than at the basal electrodes. Eight cases showed patterns of impedance deviation that were not seen in the other cases (Figure 1). We observed a long-term increase in impedances (cases 34, 41), single events of impedance spikes (case 13), and fluctuating impedances (cases 20, 24, 28, 33, 40).

### 3.3. Residual hearing progression over time

The progression of residual hearing and impedances (i.e., clinical impedance, near-field impedance, and far-field impedance) over time shows, for most of the 42 cases, a negative correlation between the two variables (see Supplementary Figures 2–4). Preoperative residual hearing from the 42 cases was in the range of 9–65 dB HL with a mean of 37 dB HL. Over time, 12 cases (29%) completely lost their residual hearing after a mean follow-up time of 25 months (ranging from 7 to 49 months). Residual hearing was still present in the remaining 30 cases (71%) at the last audiological assessment in our dataset (mean follow-up time of 35 months, ranging from 1 to 98 months). Hearing preservation after 6 months, according to (33), is shown in Supplementary Table 1.

### 3.4. Association of residual hearing and impedance subcomponents

We included a total of 152 audiological measurements and the same number of concurrent telemetry recordings from the 42 cases in the linear mixed-effects models for residual hearing. Near-field impedance had a statistically significant negative effect on residual hearing (−3.81 dB HL per kΩ, *p* < 0.001; Table 1). Postoperative follow-up time was also associated with a significant negative effect on residual hearing (−0.29 dB HL per month; *p* < 0.001). The FLEX28, FLEXSoft, and Standard electrode arrays were associated with significantly lower preoperative residual hearing than the FLEX24 electrode array (−20.46 dB HL, *p* < 0.001; −23.73 dB HL, *p* = 0.004; −33.11 dB HL, *p* = 0.01, respectively). The interaction of time with both near-field and far-field impedances showed an association with residual hearing (0.06 and −0.28 dB HL, respectively; *p* < 0.001 for both). No significant effects of far-field impedance, side, gender, age at implantation, or cochlear duct length were found.

**Table 1:** Linear mixed-effects model summary table for residual hearing (in dB HL) depending on near-field and far-field impedance including all electrodes.

	**Coefficient**	**95% CI**	***p*-value**
*Intercept*	64.12	[−0.95; 129.9]	0.08
Time (months)	−0.29	[−0.43; −0.16]	<0.001
Near-field impedance (kΩ)	−3.81	[−4.57; −3.05]	<0.001
Far-field impedance (kΩ)	1.67	[−2.55; 5.92]	0.44
Side_R_	−1.24	[−8.34; 5.87]	0.75
Gender_M_	−1.14	[−8.37; 6.13]	0.77
Age at implantation (years)	0.18	[−0.03; 0.4]	0.12
Cochlear duct length (mm)	−0.54	[−2.4; 1.29]	0.6
Electrode array_FLEX28_	−20.46	[−28.54; −12.41]	<0.001
Electrode array_FLEXSoft_	−23.73	[−37.67; −9.78]	0.004
Electrode array_Standard_	−33.11	[−55.63; −10.59]	0.01
Interaction of time with near-field impedance	0.06	[0.05; 0.07]	<0.001
Interaction of time with far-field impedance	−0.28	[−0.39; −0.16]	<0.001
Num. obs.	1,747
Num. groups: Cases	42

R, right; M, male; CI, confidence interval.

**Figure 2:**
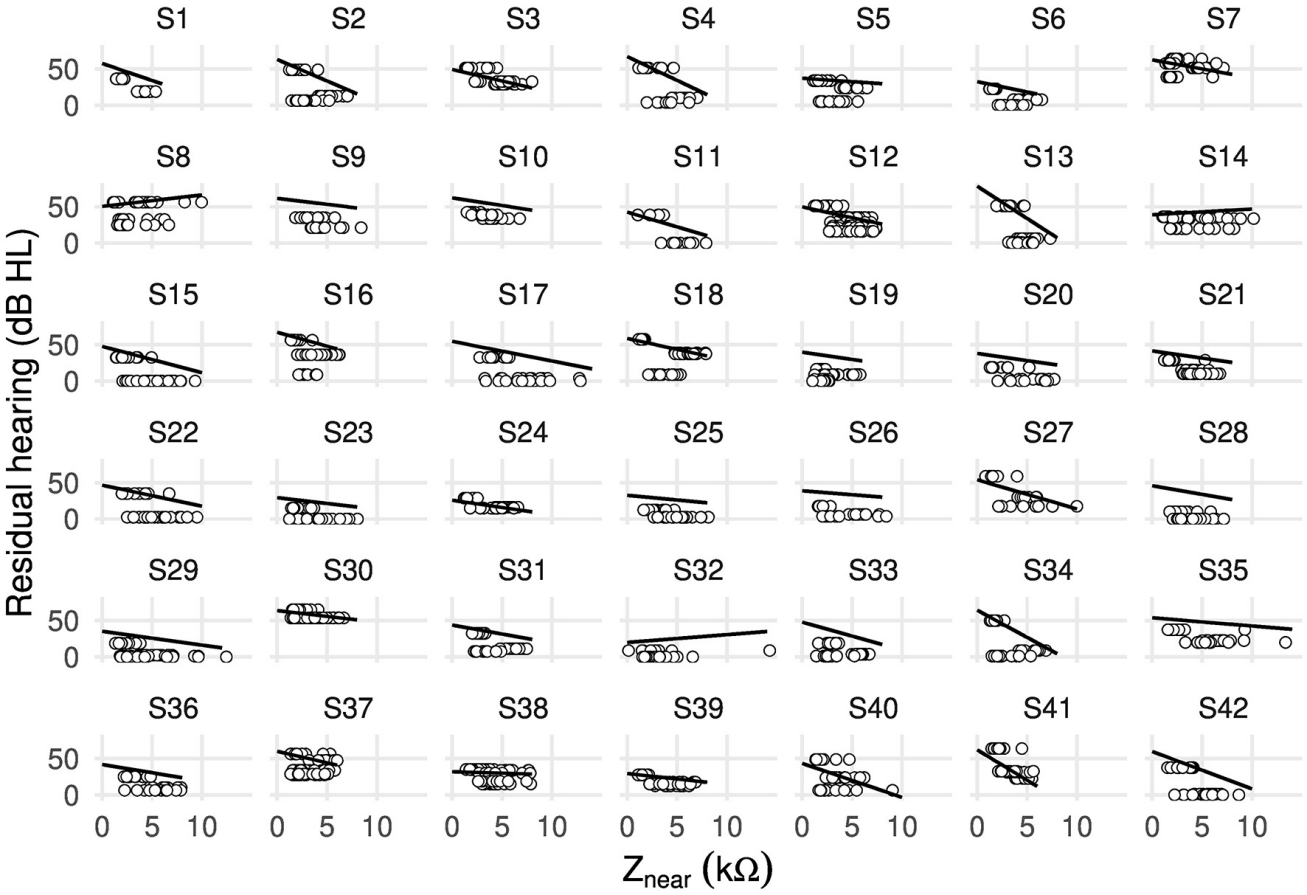
Scatter plot showing random intercepts and random slopes as estimated by the linear mixed-effects model between residual hearing (in dB HL) and near-field impedance Z_near_ (in kΩ) including all electrodes of 42 cases.

Figure 2 shows, for each case, the fitted random intercepts and slopes between residual hearing and near-field impedance. The resulting slopes (i.e., the effect of an increase in near-field impedance by 1 kΩ on residual hearing in dB HL) were negative for all cases except 8, 14, and 32.

The linear mixed-effects model depending on clinical impedance resulted in the same significant effects on residual hearing as the near and far-field model (see Supplementary Table 3). Clinical impedance was associated with a statistically significant negative effect on residual hearing (−3.8 dB HL per kΩ; *p* < 0.001). The comparison of the two linear mixed-effects models resulted in lower AIC and BIC indices of the near and far-field model compared to the clinical impedance model (see Supplementary Table 2). In addition, the conditional *R*^2^ of the near and far-field model was 2% higher (*R*^2^ = 87%), while the marginal *R*^2^ (i.e., explained variance by the fixed effects only) was the same for both models. The ICC of the near and far-field model was 3% higher compared to the clinical impedance model (ICC = 76%).

## 4. Discussion

### 4.1. Impedance subcomponents progression over time

Near-field impedances increased strongly until the first month after implantation (Figure 1), which was also observed for clinical impedances (17, 23, 34). This might be due to intracochlear inflammatory reactions or wound healing (22, 23, 35). Intracochlear trauma and fibrous tissue formation are most prominent in the basal turn (36–38). We observed this in the long-term increase of near-field impedances, especially at the two most basal electrodes (see Supplementary Figure 1). More apically located electrodes are associated with higher far-field impedances (21). On average, we found higher far-field impedances near the apex than in the basal turn (see Supplementary Figure 1).

We propose to assign the unusual patterns of near-field impedances (i.e., deviating from the initial rise in near-field impedance to CI activation and subsequent stabilization) to three groups (Figure 1). The long-term increase of near-field impedances in the first group might be due to fibrous tissue growth in the hook region (9, 36), reducing electrical conductivity near most basal electrodes. Single near-field impedance spikes or fluctuating near-field impedances in the second and third groups could be related to clinical events such as hearing loss, tinnitus, or vertigo (17). However, we found no such events in the medical records of these cases.

### 4.2. Residual hearing progression over time

Slopes of residual hearing progression over time were negative in all cases except for case 18 (see Supplementary Figure 2). Seventy-one percent of cases in our dataset had remaining residual hearing at the last audiological assessment. Our findings are comparable to Snels et al. (5), who found mean hearing preservation after 12 months or more of ~70%. However, in the presented cases with remaining residual hearing, the mean follow-up was considerably higher (35 months).

### 4.3. Association of residual hearing and impedance subcomponents

Near-field impedance showed a strong association with residual hearing (−3.81 dB HL per kΩ, Table 1). In line with our findings, Tejani et al. (39) found elevated access resistance *R*_*a*_ in cases with loss of residual hearing, while the polarization impedance *Z*_*p*_ remained stable. In our study, near-field impedance consists of the polarization impedance *Z*_*p*_ and the bulk resistance *R*_*b*_ (*R*_*b*_ is part of the access resistance *R*_*a*_). This contrasts with Leblans et al. (16), who defined near and far-field impedances as subcomponents of the access resistance *R*_*a*_. Near-field impedance had approximately the same effect on residual hearing as clinical impedance. This is because the far-field impedance remained relatively stable over time, and most impedance fluctuations were due to the near-field impedance. The model depending on impedance subcomponents was better than the model depending on clinical impedance because the former explained more variance in residual hearing (conditional *R*^2^ = 87%). Because the improvement in explaining variance is small (2%), the subcomponent model does not provide substantially better performance on group level. However, the model can explicitly demonstrate the isolated contributions to long-term variation of the near- and far-field components and serve as a basis for further investigation. In this context, we consider near-field impedance to be most clinically relevant. In addition, individual long-term variations in far-field impedances can be isolated that could otherwise have affected the accuracy of the model.

We included electrode array type and cochlear duct length as fixed effects in the model. We observed a preference for shorter electrode arrays to preserve a comparatively high preoperative residual hearing (see Supplementary Figure 5). The active stimulation range (i.e., the array's length from the middle of the first to the middle of the last electrode) is shortest for FLEX24 (20.9 mm), followed by FLEX28 (23.1 mm) and FLEXSoft, Standard (26.4 mm). However, the sample size in our dataset was not evenly distributed among the different electrode array types (26 × FLEX28, 12 × FLEX24, 3 × FLEXSoft, and 1 × Standard). The distribution of preoperative residual hearing was wider for the FLEX28 electrode array than for the other. A longer cochlea does not appear to have influenced the choice of electrode array type in our dataset (see Supplementary Figure 6). Therefore, we kept electrode array type and cochlear duct length as fixed effects.

For the model, we did not include pure tone frequency as a fixed effect. Therefore, we created a separate model for residual hearing with pure tone frequency (categorical, in kHz) as an additional fixed effect (i.e., modeling residual hearing at distinct frequencies instead of low-frequency PTA). The model resulted in the same significant effects on residual hearing, except for the FLEXSoft electrode array (see Supplementary Table 4). The effect of near-field impedance on residual hearing was approximately 1 dB HL smaller than in the model with low-frequency PTA as the dependent variable. As expected, pure tone frequency had a significant effect on residual hearing. This was expected because residual hearing varies with position in the cochlea and is best preserved over time in the apical region (40). Preoperative residual hearing was highest at 0.25 kHz and not at the lowest frequency of 0.125 kHz.

In contrast to Wimmer et al. (23), we did not divide the electrodes into subgroups for our analysis of residual hearing and impedance telemetry data. While the low-frequency range from 0.125 to 1 kHz corresponds to the most apical electrodes [insertion depth of ~360–720°, (41)], Wimmer et al. (23) found that the most significant effect on residual hearing is observed in the impedance data of the most basal electrodes. Fibrous tissue and new bone formation in the cochlea resulting from electrode array insertion could reduce basilar membrane compliance not only near the round window (8) but also at multiple locations along the entire auditory pathway from the oval window to the apex. Based on this, we decided to include data from all electrodes in our analysis.

During model checks, we observed collinearities between independent variables. High Variance inflation factors (VIFs) were present for the near-field impedance (VIF = 1.03, tolerance = 0.98) and the interaction between follow-up time and far-field impedance (VIF = 46.86, tolerance = 0.02). As VIF might be inflated in the presence of interaction terms (42), we checked multicollinearity among independent variables without interaction terms. This resulted in only a low correlation among independent variables.

### 4.4. Study limitations and outlook

We could not measure near and far-field impedances directly but estimated them from the recorded impedance matrix using an approximation model (21). Estimated impedance subcomponents could therefore have included contributions from the complementary subcomponent and vice versa. Ultimately, this could have introduced modeling errors for residual hearing using impedance subcomponents. Advanced measurement techniques (43) could overcome this limitation and measure directly the polarization impedance (as part of the near-field impedance) and access resistance (which includes the far-field impedance). Our analysis was based on only 152 audiological measurements from 42 cases. Future studies need to verify our results in a larger dataset.

This study did not investigate the influence of electrode insertion depth on residual hearing. As electrode location is a potential biomarker for intracochlear trauma and hearing preservation (3), this could improve the prediction accuracy for residual hearing.

## 5. Conclusion

In this study, we showed that near-field impedance is strongly associated with postoperative residual hearing. This supports our hypothesis that impedance subcomponents provide a more specific analysis of the cochlear microenvironment and hearing performance compared with conventional impedance telemetry. As far-field impedance was not associated with changes in residual hearing, we concluded that residual hearing is primarily influenced by the local cochlear microenvironment around the electrode contacts. To further improve modeling accuracy for residual hearing, future studies will need to use advanced measurement techniques for direct measurement of impedance subcomponents.

## Data availability statement

The original contributions presented in the study are included in the article/Supplementary material, further inquiries can be directed to the corresponding author.

## Ethics statement

The studies involving human participants were reviewed and approved by Kantonale Ethikkommission Bern Universität Bern (ID 2019-01578). Written informed consent to participate in this study was provided by the participants' legal guardian/next of kin.

## Author contributions

SS: methodology, software, formal analysis, visualization, and writing—original draft. PA: methodology and writing—review and editing. SW: data curation and writing—review and editing. MC: resources and writing—review and editing. WW: conceptualization, methodology, formal analysis, writing—review and editing, project administration, and funding acquisition. All authors contributed to the article and approved the submitted version.
