# Supplementary material for: Cochlear implant electrode impedance subcomponents as biomarker for residual hearing
Source: Front Neurol. 2023 May 23;14:1183116. doi: 10.3389/fneur.2023.1183116 (PMC10242064; doi:10.3389/fneur.2023.1183116)
Supplement: Supplementary file 1 [file Data_Sheet_1.PDF]

Supplementary Material

1 SUPPLEMENTARY TABLES AND FIGURES

1.1 Figures

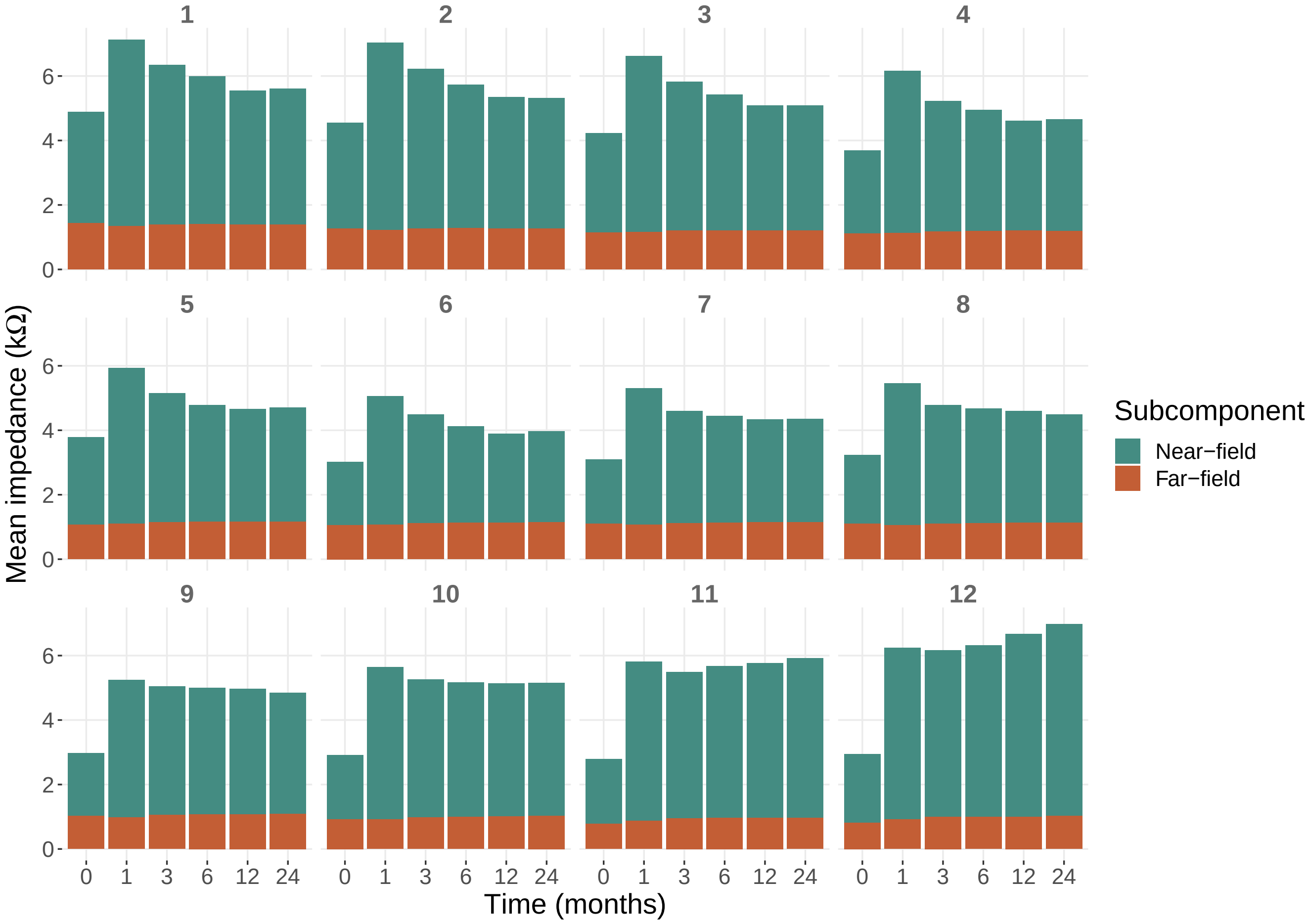

Figure S1. Stacked bar plot of mean impedance subcomponents per electrode over time. Electrodes are numbered from apical to basal (1-12, starting top left). Data are from 42 cases at the intraoperative measurement (month 0), first activation session (month 1), and follow-up fitting sessions (months 3-24).

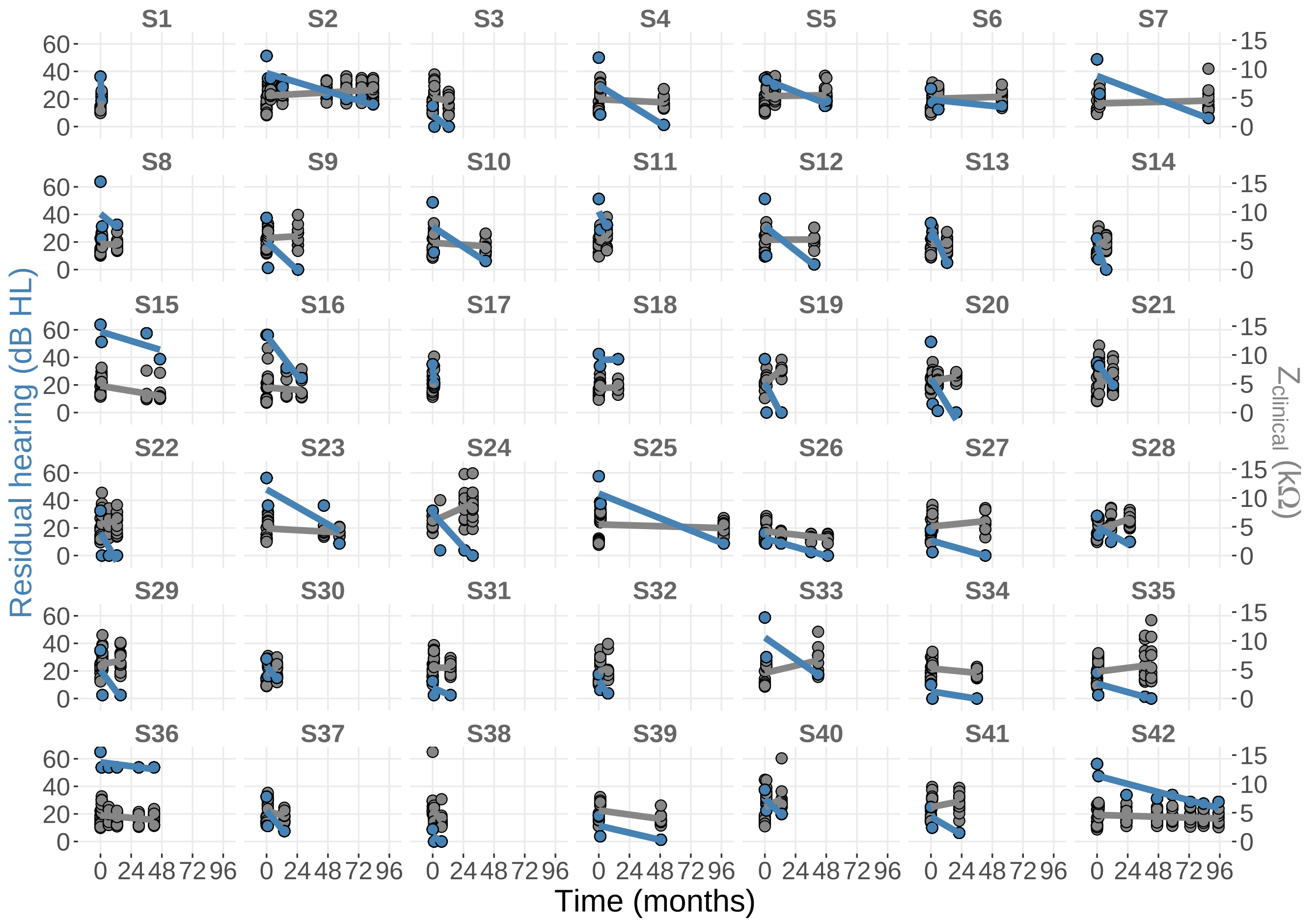

Figure S2. Scatter plot of residual hearing (in dB HL) and clinical impedance Z_{clinical} (in kΩ) over time of 42 cases including all electrodes. Straight lines indicate linear regression lines.

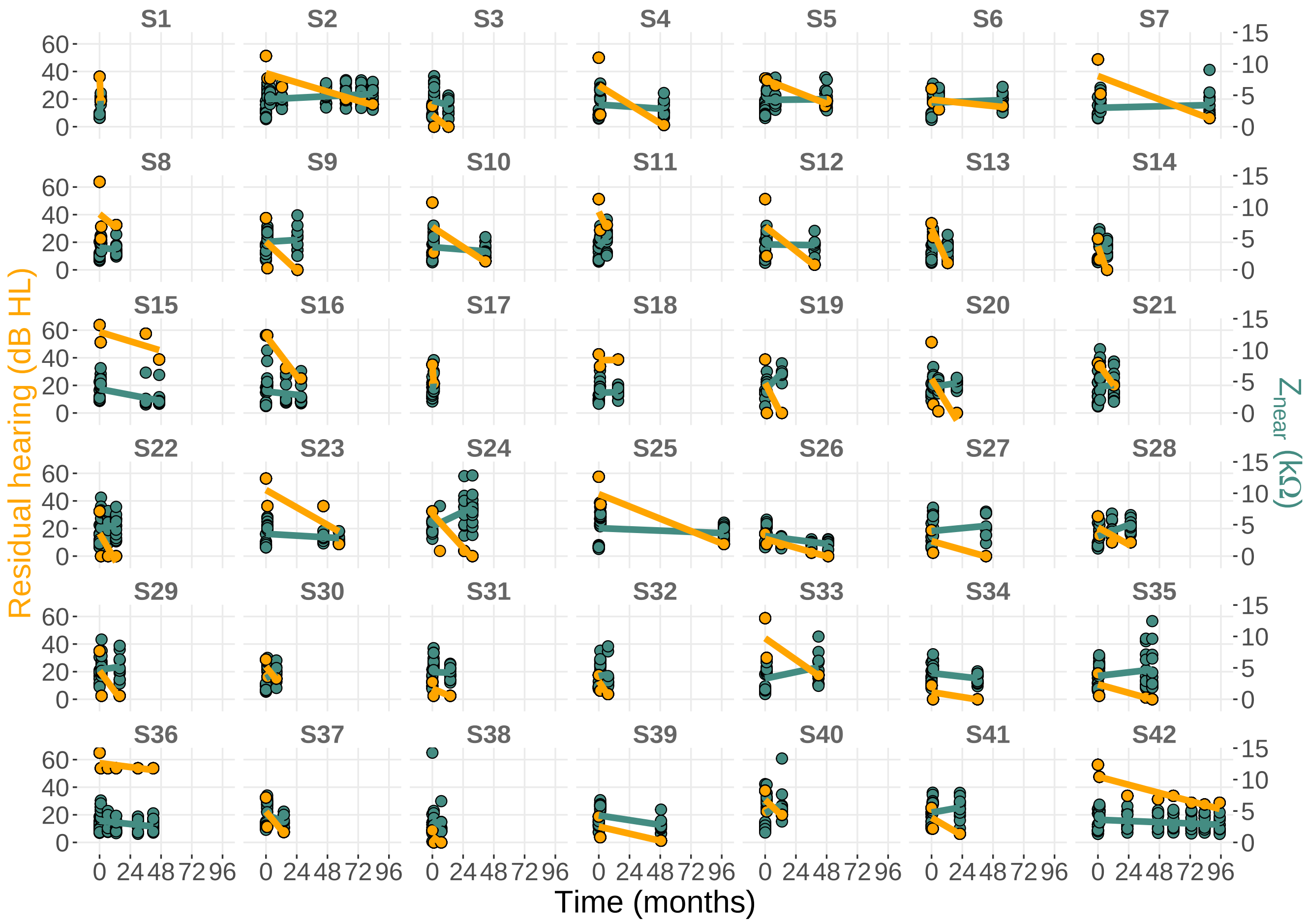

Figure S3. Scatter plot of residual hearing (in dB HL) and near-field impedance Z_{near} (in $k\Omega$) over time of 42 cases including all electrodes. Straight lines indicate linear regression lines.

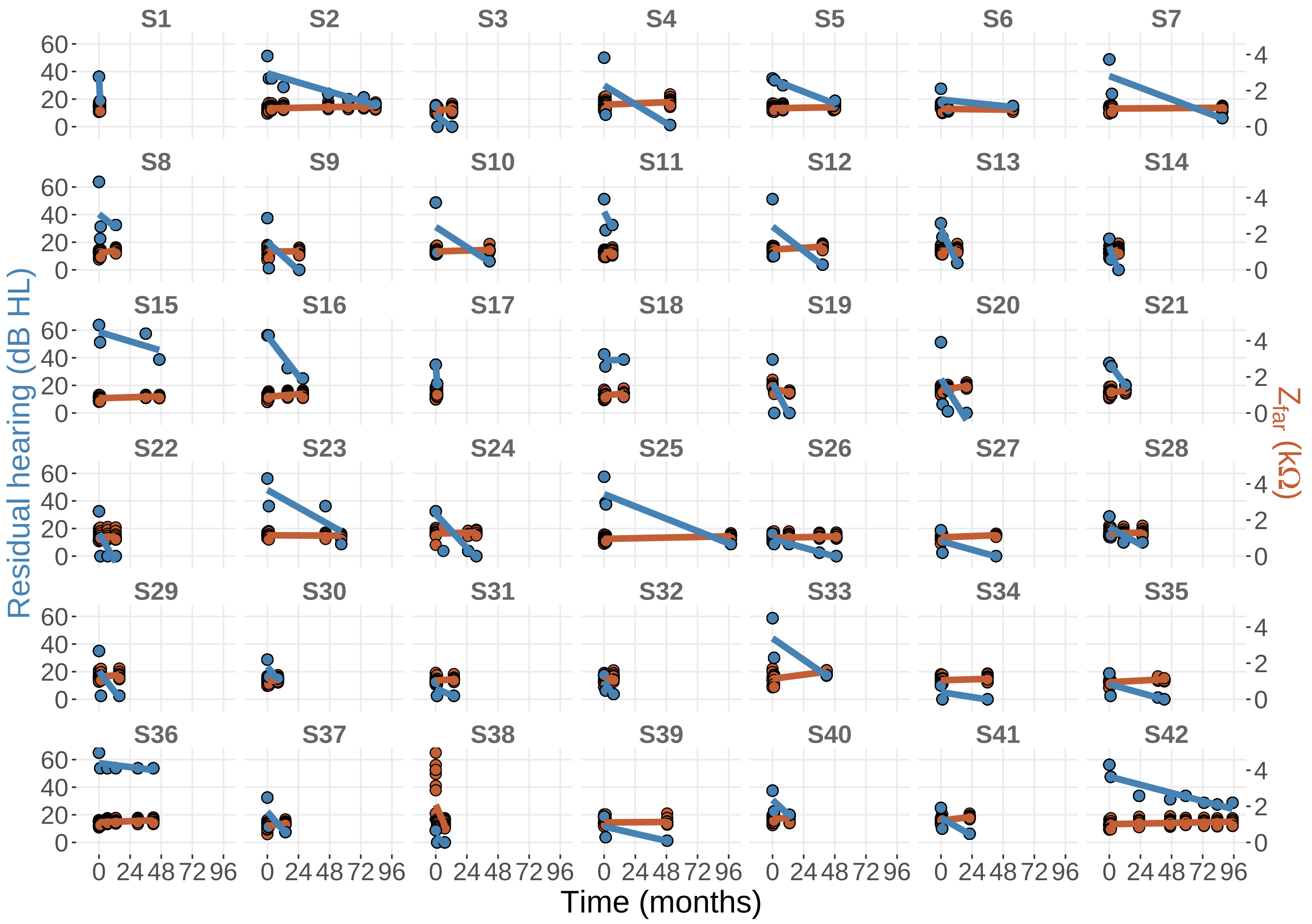

Figure S4. Scatter plot of residual hearing (in dB HL) and far-field impedance Z_{far} (in kΩ) over time of 42 cases including all electrodes. Straight lines indicate linear regression lines.

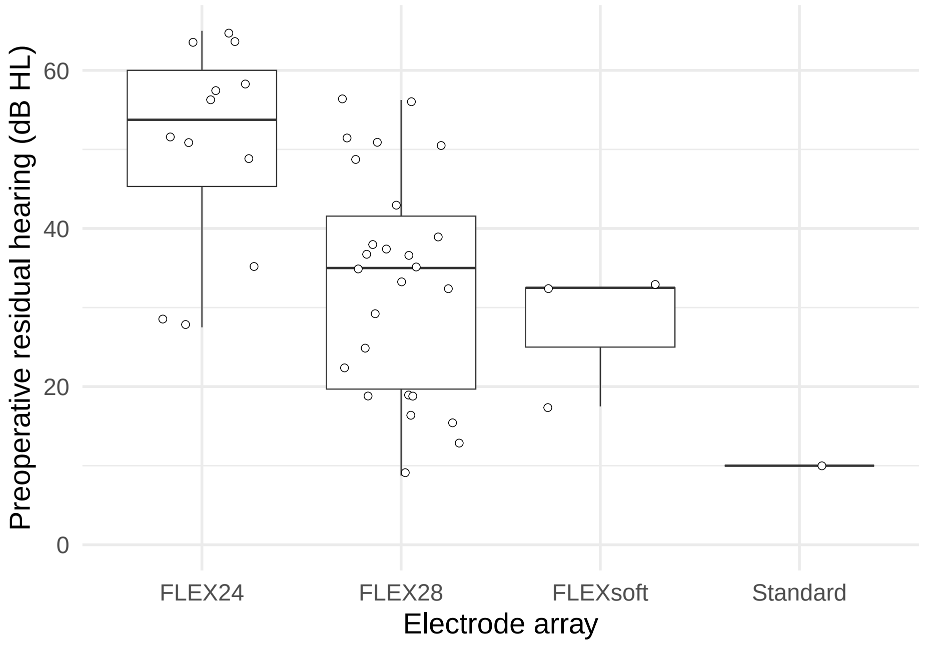

Figure S5. Preoperative residual hearing (in dB HL) versus electrode array type of 42 cases.

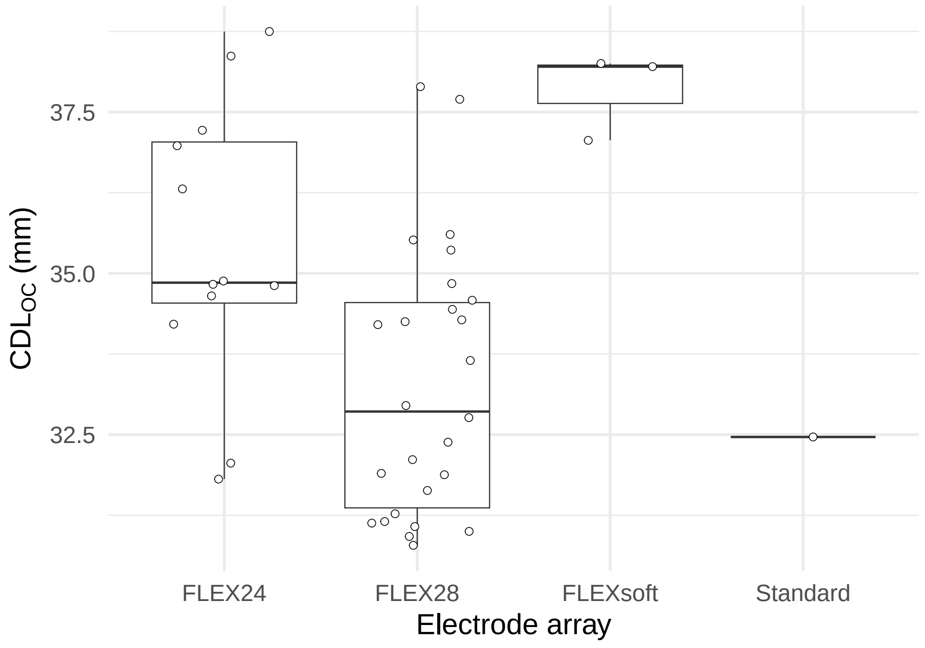

Figure S6. Cochlear duct length (in mm) versus electrode array type of 42 cases.

1.2 Tables

Table S1. Demographic details of dataset and functional outcomes, sorted by relative preservation of residual hearing after 6 months. F, female; M, male; R, right; L, left.

ID	Gender	Age	Side	Etiology	Follow-up	Electrode array	Insertion	Word recognition scores*		Hearing preservation**		
		(years)			(months)			Monosyllables (%)	Numbers (%)	Absolute (dB HL)	Relative (%)	Category
5	F	57	R	Progressive	49	FLEX^{24}	Full	100	100	-5	86	*1*
36	M	60	R	Progressive	41	FLEX^{24}	Full	40	90	-4	83	*1*
18	F	56	L	Progressive	15	FLEX^{28}	Full	50	100	-8	82	*1*
15	M	69	L	Progressive	47	FLEX^{24}	Full	70	100	-13	80	*1*
11	F	61	R	Progressive	6	FLEX^{24}	Full	50	90	-15	65	*2*
23	F	67	L	Progressive	58	FLEX^{28}	Partial (9 of 12)	65	100	-20	65	*2*
2	F	55	L	Progressive	84	FLEX^{24}	Full	95	100	-19	63	*2*
42	F	53	R	Progressive	94	FLEX^{24}	Full	100	100	-23	61	*2*
16	F	59	L	N/A	28	FLEX^{28}	Full	85	100	-24	59	*2*
40	F	24	L	Progressive	13	FLEX^{28}	Full	45	100	-10	59	*2*
17	M	70	R	Progressive	14	FLEX^{28}	Full	80	100	-14	58	*2*
26	F	26	L	Progressive	49	FLEX^{28}	Full	95	100	-8	56	*2*
6	F	43	R	Progressive	56	FLEX^{24}	Full	10	100	-10	56	*2*
30	F	39	L	Progressive	8	FLEX^{24}	Full	75	100	-13	55	*2*
21	F	80	R	Progressive	13	FLEX^{28}	Full	40	100	-16	55	*2*
13	M	74	L	Sudden	13	FLEX^{28}	Full	100	100	-17	48	*2*
8	M	60	R	Progressive	13	FLEX^{24}	Full	65	100	-31	48	*2*
28	F	43	L	Progressive	25	FLEX^{28}	Full	40	40	-19	40	*2*
33	F	52	R	Progressive	41	FLEX^{24}	Partial (10 of 12)	70	90	-41	31	*2*
41	F	73	R	Hydrops	22	FLEX^{28}	Full	20	80	-19	25	*2*
31	F	32	R	Progressive	21	FLEX^{28}	Full	45	80	-6	23	*3*
37	F	40	L	Progressive	13	FLEX^{28}	Full	45	80	-25	23	*3*
32	F	39	R	Progressive	7	FLEX^{Soft}	Full	75	100	-14	22	*3*
25	M	26	L	Progressive	104	FLEX^{24}	Full	85	90	-49	16	*3*
10	F	61	R	Progressive	42	FLEX^{28}	Full	65	100	-41	15	*3*
7	M	71	L	Progressive	88	FLEX^{24}	Full	25	100	-43	13	*3*
24	F	62	L	Progressive	31	FLEX^{Soft}	Full	65	100	-29	10	*3*
12	F	70	R	Sudden	39	FLEX^{28}	Partial (9 of 12)	50	80	-48	10	*3*
29	F	44	R	Progressive	15	FLEX^{28}	Full	40	60	-30	9	*3*
35	M	69	R	Progressive	42	FLEX^{28}	Full	85	100	-19	5	*3*
1	M	61	R	Progressive	10	FLEX^{28}	Full	45	90	-34	5	*3*
20	M	63	R	Sudden	20	FLEX^{28}	Full	25	90	-50	3	*3*
4	M	71	L	Progressive	52	FLEX^{28}	Full	20	40	-49	2	*3*
39	M	45	L	Progressive	48	FLEX^{28}	Full	0	20	-19	1	*3*
34	M	52	L	Trauma	35	Standard	Full	80	100	-10	0	*4*
22	M	64	R	Hydrops	13	FLEX^{Soft}	Full	75	100	-33	0	*4*
38	M	42	R	Trauma	6	FLEX^{28}	Full	70	100	-9	0	*4*
9	M	46	R	Progressive	25	FLEX^{28}	Full	70	100	-38	0	*4*
3	M	59	L	Progressive	13	FLEX^{28}	Full	50	100	-15	0	*4*
27	F	35	L	Progressive	42	FLEX^{28}	Partial (9 of 12)	50	100	-19	0	*4*
19	M	52	L	Hydrops	13	FLEX^{28}	Partial (6 of 12)	20	50	-39	0	*4*
14	F	61	L	Meningitis	7	FLEX^{28}	Partial (11 of 12)	0	60	-23	0	*4*

*German Freiburg monosyllabic word lists at 65 dB sound pressure level (SPL) and German Freiburg numbers at 60 dB SPL after 6 months.
**Residual hearing category according to Skarzynski et al. after 6 months.

Table S2. Comparison of model performance indices of the linear mixed-effects models for residual hearing depending on clinical impedance and depending on near-field together with far-field impedance including all electrodes. AIC, Akaike information criterion; BIC, Bayesian information criterion; R^2, coefficient of determination; ICC, Intraclass correlation coefficient.

	**AIC**	**BIC**	R^2 **(cond.)**	R^2 **(marg.)**	**ICC**
Clinical model	12240	12322	0.85	0.46	0.73
Near and far-field model	12178	12287	0.87	0.46	0.76

Table S3. Linear mixed-effects model summary table for residual hearing (in dB HL) depending on clinical impedance including all electrodes. R, right; M, male; CI, confidence interval.

	**Coefficient**	**95% CI**	**p-value**
Intercept	75.31	[11.51; 139.2]	.04
Time (months)	-0.65	[-0.71; -0.6]	<.001
Clinical impedance (kΩ)	-3.8	[-4.57; -3.04]	<.001
Side_{R}	-1.59	[-8.62; 5.45]	.69
Gender_{M}	-0.94	[-7.99; 6.1]	.8
Age at implantation (years)	0.18	[-0.03; 0.4]	.12
Cochlear duct length (mm)	-0.7	[-2.51; 1.11]	.49
$\text{Electrode array}_{\text{FLEX28}}$	-20.11	[-28.06; -12.18]	<.001
$\text{Electrode array}_{\text{FLEXSoft}}$	-23.12	[-36.96; -9.28]	.005
$\text{Electrode array}_{\text{Standard}}$	-34.39	[-56.78; -12.01]	.01
Interaction of time with clinical impedance	0.06	[0.05; 0.07]	<.001
Num. obs.	1747		
Num. groups: Cases	42		

Table S4. Linear mixed-effects model summary table for residual hearing (in dB HL) with frequency (categorical, in kHz) as additional fixed effect including all electrodes. R, right; M, male; CI, confidence interval.

	**Coefficient**	**95% CI**	**p-value**
Intercept	47.2	[-6.14; 101.1]	.11
Time (months)	-0.12	[-0.22; -0.02]	.02
Near-field impedance (kΩ)	-2.82	[-3.4; -2.24]	<.001
Far-field impedance (kΩ)	2.63	[-1.72; 7]	.24
Side_{R}	-1.44	[-7.06; 4.18]	.65
Gender_{M}	-0.66	[-6.58; 5.27]	.83
Age at implantation (years)	0.08	[-0.09; 0.25]	.4
Cochlear duct length (mm)	-0.17	[-1.68; 1.32]	.83
$\text{Electrode array}_{\text{FLEX28}}$	-11	[-17.57; -4.49]	.004
$\text{Electrode array}_{\text{FLEXSoft}}$	-12.07	[-23.08; -1.06]	.06
$\text{Electrode array}_{\text{Standard}}$	-20.75	[-38.67; -2.84]	.04
$\text{Frequency}_{0.25}$	6.94	[6.03; 7.85]	<.001
$\text{Frequency}_{0.5}$	-1.5	[-2.41; -0.58]	.001
Frequency_{1}	-13.3	[-14.24; -12.41]	<.001
Frequency_{2}	-17.3	[-18.25; -16.42]	<.001
Frequency_{4}	-17.6	[-18.54; -16.71]	<.001
Frequency_{8}	-20.8	[-21.67; -19.85]	<.001
Interaction of time with near-field impedance	0.05	[0.04; 0.05]	<.001
Interaction of time with far-field impedance	-0.3	[-0.38; -0.21]	<.001
Num. obs.	12229		
Num. groups: Cases	42		
